# Endophthalmitis following intravitreal anti-vascular endothelial growth factor (VEGF) injection: a comprehensive review

**DOI:** 10.1186/s40942-015-0010-y

**Published:** 2015-07-21

**Authors:** Rohan Merani, Alex P Hunyor

**Affiliations:** 1 Retina Associates, Level 4, 8 Thomas St, Chatswood, NSW 2067 Australia; 2 Save Sight Institute, University of Sydney, Sydney, NSW Australia; 3 Australian School of Advanced Medicine, Macquarie University, Sydney, NSW Australia; 4 Concord Repatriation General Hospital, Concord, NSW Australia; 5 Sydney Eye Hospital, Sydney, NSW Australia

**Keywords:** Endophthalmitis, Intravitreal injection, Anti-VEGF, *Streptococcus*, Masks, Antisepsis, Povidone-Iodine, Chlorhexidine, Antibiotics, Speculum

## Abstract

The purpose of this review is to report and summarize previously reported studies and assess many of the individual steps of the intravitreal injection procedure’s possible effect on the prevention of endophthalmitis. The pooled endophthalmitis rate from 20 large retrospective case series of anti-VEGF injections was 144/510,396 (0.028%; 1/3,544). Injections may be performed in an office-based location or in an operating room (OR) and low rates of endophthalmitis can be achieved in either location with careful attention to asepsis. Pre- or post-injection topical antibiotics have not been shown to be effective, and could select for more virulent microorganisms. Povidone-iodine prior to injection is accepted as the gold-standard antiseptic agent, but *aqueous* chlorhexidine may be an alternative. Antisepsis before and after gel or subconjunctival anesthetic is suggested. The preponderance of Streptococcal infections after intravitreal injection is discussed, including the possible role of aerosolization, which can be minimized by using face masks or maintaining silence. As with other invasive procedures in medicine, the use of sterile gloves, following adequate hand antisepsis, may be considered. Control of the eyelashes and lid margin is required to avoid contamination of the needle, but this can be achieved with or without a speculum. Techniques to minimize vitreous reflux have not been shown to reduce the risk of endophthalmitis. Same day bilateral injections should be performed as two separate procedures, preferably using drug from different lots, especially when using compounded drugs.

## Introduction

Intravitreal injection (IVI) is the most commonly performed ophthalmic procedure. In the USA, the number of injections performed has increased exponentially, from 4,215 injections in 2001 to 82,994 in 2004, to 812,413 in 2007, to 1.27 million in 2009 and to 2.5 million injections in 2011 [1, 2]. Similar increases have been observed in Canada and the United Kingdom [3, 4].

Infectious endophthalmitis (IE) secondary to IVI is a potentially devastating complication. It can be difficult to distinguish infectious endophthalmitis from “sterile” or non-infectious endophthalmitis. For the purpose of this review, IE refers to endophthalmitis that is clinically suspected to be infectious, and treated as such with a vitreous tap and injection of antibiotics and/or vitrectomy surgery.

Bacteria are most likely inoculated into the vitreous cavity at the time of injection, or much less likely gain access later through the needle tract [5, 6]. The potential sources of bacteria include the patient’s ocular or periocular surfaces, aerosolized bacteria, or contamination of the needle, instruments, drug or drug vial [7].

Two meta-analyses including both retrospective series and clinical trials have calculated the pooled rate of endophthalmitis after anti-VEGF injections. McCannel found a rate of 52/105,536 injections (0.049%; 1 in 2030) [8] and more recently, Fileta et al. [9] calculated a rate of 197/350,535 (0.056%; 1 in 1,779). As patients typically receive ongoing intravitreal therapy, the per-patient risk of endophthalmitis is significantly higher than the per-injection risk.

The rate of needle contamination after IVI has been reported as between 0.36 and 18%, which is significantly higher than the incidence of endophthalmitis after IVI [5, 7, 10]. The threshold inoculum size required to develop endophthalmitis is related to the type of bacteria and their virulence, intraocular immune mechanisms and anatomical characteristics of the vitreous [11, 12]. Animal studies have shown that a smaller number of bacterial colony-forming units are required to induce endophthalmitis when injected into the vitreous compared to when they are injected into the anterior chamber [13]. Endophthalmitis following intravitreal injection often presents earlier than after cataract surgery [14, 15].

The purpose of this review is to estimate the rate of endophthalmitis after intravitreal injection and to examine each step of the injection procedure that may influence the risk of endophthalmitis. To be able to prove that a particular measure reduces the risk of endophthalmitis would need huge numbers of patients in a randomized controlled trial, given that endophthalmitis is a relatively rare outcome. There is thus no Level 1 evidence for any preventative measure to reduce the incidence of endophthalmitis after intravitreal injection. As a result, this review largely summarizes retrospective papers, with their inherent biases.

## Methods

A systematic literature search of the Medline database from 1996 to December 2014 was performed through Ovid, using search terms relevant to each section. Further literature was sourced from the reference lists of retrieved publications.

To estimate the per-injection rate of endophthalmitis after anti-VEGF injection, retrospective case series with at least 10,000 such injections were included. Studies that did not report a breakdown of the drugs used were excluded to avoid including triamcinolone and other injections in this calculation. Questionnaire-based and population-based studies were excluded given the incomplete data. Clinical trials were excluded as they may not reflect real-world practice, with more stringent requirements regarding injection technique often included in the protocols.

## Results

Twenty retrospective case series meeting the inclusion criteria were identified. Details of the injection procedure and associated aseptic measures used in each series are summarized in the Table 1. Where data were missing, the corresponding author for each study was contacted by email. Only two authors were not contactable.

**Table 1:** Endophthalmitis following intravitreal anti-VEGF injection—retrospective cases series with at least 10,000 injection

Period and location	Authors	n = injections	Rate of clinically suspected IE	Pre-injection antibiotics	Post injection antibiotics	Mask	Drape	Conjunctival povidone-iodine concentration	Anaesthetic agents used	Sterile lid speculum	Gloves	Location
1 Jan 2009 to 1 Oct 2012 Single-centre, USA	Storey et al. [16] (PIE study group) Ophthal 2013	117,171	**Overall** 44/117,171 (0.038%; 1/2,663) **By Agent** Rani: 24/71,791 (0.033%; 1/2,991) Bev: 20/44,007 (0.045%;1/2,200) Aflib: 0/1,373	Variable	Variable	No*	No	5%	Drops Subconj (rarely)	Variable	Nil*	Office
1 Jan 2005 to 31 Dec 2010 Single-centre (multi-site), USA	Moshfeghi et al. [17] Retina 2011	60,322	**Overall** 12/60,322 (0.020%; 1/5,027) **By Agent** Rani: 5/18,607 (0.027%; 1/3,721) Bev: 7/39,700 (0.018%; 1/5,671) Peg: 0/2,015	No	Variable	No*	No	5%	Drops	Yes	Non-sterile	Office
1 Jan 2007 to 31 Dec 2011 Single-centre, USA	Chaudhary et al. [18] Retina 2013	49,002	**Overall** 17/49,002 (0.035%; 1 in 2,882) **By Agent** Rani: 2/20,297 (0.0099%;1/10,149) Bev: 15/28,705 (0.052%;1/1,914)	Yes	Yes	No	No	5%	Drops Gel Subconj	Yes	Nil or non-sterile	Office
2004 to 2012 Multicentre, Switzerland	Casparis et al. [19] Retina 2014	40,011	**Overall** 3/40,011 (0.0075%;1 in 13,337) **By Agent** Rani: 3/36,398 (0.0082%, 1/12,133) Bev: 0/3,518 Aflib:0/89 Peg: 0/6	No	Variable (yes in one hospital, no in the other)	Yes	Yes (adhesive)	5–10%	Drops	Yes	Sterile	OR
1 Aug 2006 to 31 Jul 2007 Multi-centre, USA	Klein et al. [20] Ophthal 2009	30,736	**Overall** 15/30,736 (0.049%; 1/2,049) **By Agent** Rani: 10/22,579 (0.044%; 1/2,258) Bev: 5/8,039 (0.062%; 1/1,608) Peg: 0/128	Variable	?	?	?	5–10%	?	Variable: used in 14 of the 15 cases with endophthalmitis	?	Office
July 2000 to July 2010 Single-centre (multi-site), USA	Chen et al. [21] Retina 2011	29,995	**Overall** 11/29,995 (0.037%; 1/2,727) **By Agent** Rani: 8/22,336 (0.036%; 1/2,792) Bev: 3/6,675 (0.045%; 1/2,225) Peg: 0/984	Yes	Yes	No*	No*	5–10%	Drops Gel Subconj	Yes	Non-sterile*	Office
1 Jun 2005 to 7 Aug 2007 Multicentre, USA	Fintak et al. [22] Retina 2008	26,905	**Overall** 6/26,905 (0.022%; 1/4,484) **By Agent** Rani: 3/14,320 (0.021%; 1/4,773) Bev: 3/12,585 (0.024%; 1/4,195)	No*	Variable (used in all the cases of endophthalmitis)	No*	No*	5–10%	Drops Gel Subconj	Variable (used in all the cases of endophthalmitis) Fingers to spread the eyelids in a minority	Nil*	Office
March 2007 to May 2013 Single-centre, Denmark	Brynskov et al. [23] Retina 2014	20,293	**Overall** 0/20,293 **By Agent** Rani: 0/20,024 Aflib: 0/269	No	Variable	Yes	Yes (adhesive)	5%	Drops	Yes	Sterile	OR
1 Aug 1997 to 31 Oct 2012 Single-centre, USA	Bhavsar and Sandler [24] Retina 2015	17,666	**Overall** 1/17,666 (0.0057%, 1/17,666) **By Agent** Rani: 0/1,669 Bev: 1/15,479 (0.0065%, 1/15,479) Aflib: 0/148 Peg: 0/370	No	No	No	No	5% (before and after injection)	Drops	Yes	Non-sterile	Office
Jan 2005 to end July 2012 Single-centre, Germany	Nentwich et al. [25] Retina 2014	18,202	**Overall** 3/18,202 (0.016%; 1/6,067) **By Agent** Rani: 1/10,097 (0.010%, 1/10,097) Bev: 2/7,865 (0.025%;1/3,932) Peg: 0/240	No	Yes	Yes	Yes (non-adhesive)*	1%	Drops	Yes	Sterile	OR
Jan 2007 to May 2012 Multicentre, India	Mithal et al. [26] BJO 2013	15,925	**Overall** 8/15,925 (0.050%;1/1,991) **By Agent** Rani: 1/705 (0.14%; 1/705) Bev: 7/15,035 (0.044%;1/2,275) Peg: 0/185	No*	Yes*	Yes*	Variable	2.5%*	Drops*	Yes*	Sterile*	Office*
July 2009 to July 2012 Single-centre, Japan	Shimada et al. [27] Graefes 2013	15,144	**Overall** 0/15,144 **By Agent** Rani: 0/13,750 Bev: 0/846 Peg: 0/548	Yes	Yes	Yes	Yes (adhesive)	0.25% (before and after injection)	Drops + Subconj	Yes	Yes	Office
Jan 2005 to Aug 2010 Multi-centre, Canada	Cheung et al. [28] Ophthal 2012	14,960	**Overall** 7/14,960 (0.047%; 1/2,137) **By Agent** Rani: 3/9,453 (0.032%; 1/3,151) Bev: 4/5,386 (0.074%; 1/1,347). Peg: 0/121	Variable	Variable	No*	No	10%	Drops Gel	Yes	Nil*	Office
Mar 2006 to Mar 2012 Multi-centre (single-surgeon), Australia	Abell et al. [29] BJO 2012	12,249	**Overall** 4/12,249 (0.033%; 1/3,062) **By Agent** Rani: 3/10,574 (0.028%; 1/3,525)* Bev: 1/1,675 (0.060%; 1/1,675)*	No	Variable (used until 2011)*	Yes	Yes (non-adhesive)*	10%	Drops + Gel*	Yes	Sterile	Office = 4/3,376 OR = 0/8,873
Jan 2009 to Dec 2011 Multi-centre, USA and Italy	Tabandeh et al. [30] Retina 2014	11,257	**Overall** 5/11,257 **By Agent** Rani: 3/2,724 (0.11%; 1/908) Bev: 2/8,533 (0.023%; 1/4,267)	Yes in OR No in office	Yes	Yes in OR No in office	Yes in OR No in office	5%	Drops Subconj	Yes	Sterile in OR Non-sterile in office	Office = 3/8,647 OR = 2/3,063
5 Jan 2005 to 18 Oct 2007 Single-centre (multi-site), USA	Pilli et al. [31] AJO 2008	10,254	**Overall** 2/10,254 (0.020%; 1/5,127) **By Agent** Rani: 1/6,347 (0.016%; 1/6,347) Bev: 1/3,501 (0.029%; 1/3,501) Peg: 0/406	No	Yes	No	No	5%	Drops	Variable	? “Surgical attire was not used”	Office
Nov 2010 to Dec 2011 Single-centre, USA	Fineman et al. [32] Retina 2013	10,164	**Overall** 3/10,164 (0.030%; 1/3,388) **By Agent** Rani: 1/6,330 (0.016%;1/6,330) Bev: 2/3,834 (0.052%; 1/1,917)	No*	Variable (used until Oct 2,011)	No*	No*	5%	Drops	No	Nil or non-sterile*	Office*
1 Jan 2007 to 31 Dec 2011 Single-centre, USA	Englander et al. [33] BJO 2013	10,140	**Overall** 3/10,140 (0.030%, 1/3,380) **By Agent** Rani: 3/7,768 (0.039%; 1/2,589) Bev: 0/2,315 Peg: 0/57	Variable*	Variable	Variable*	Variable	Yes, 5%	Drops Subconj.	Variable	Variable*	Office*

Steroid and other injections have been excluded.

Anesthetic: “Drops” refers to drops given alone or with the aid of a cotton bud or pledget.

Location: “office-based” includes studies where the injections were performed in a clean procedure room, or in the consulting room itself, within an office or outpatients department.

*Rani* ranibizumab, *Bev* bevacizumab, *Aflib* aflibercept and *Peg* pegaptanib.

* These points were not explicitly mentioned in the manuscript, but were clarified through personal communication with the corresponding author of the paper.

? These points were not mentioned in the manuscript, and the corresponding authors could not be contacted.

We identified 144 cases of endophthalmitis from 510,396 anti-VEGF injections which equates to a pooled endophthalmitis rate of 0.028% or 1 in 3,544 injections [16–33].

## Review

### Location—office vs operating room (OR)

In the 2013 American Society of Retinal Specialists (ASRS) Preferences and Trends (PAT) Survey, over 98% of USA-based specialists reported performing injections in an office setting, compared with only 47% of international specialists [34]. In Germany and other parts of Europe, more injections are performed in the operating room (OR) [35, 36].

It has been [29] suggested that an advantage of the OR location is the superior air circulation systems. However, the ESCRS endophthalmitis study group was not able to find a relationship between the number of air changes per hour and the incidence of endophthalmitis after cataract surgery when they compared locations with minimal airflow, 20 air changes per hour and ultraclean air systems using laminar flow principles [37, 38].

Pooling the results of three OR-based injection series, the endophthalmitis rate was just 6/78,506 (0.0076% or 1 in 13,084) [19, 23, 25]. Common to these studies was the careful attention to asepsis with the use of sterile gloves, face masks, and drapes which were not used in most other office-based series (see Table 1). A notable exception is Shimada et al’s series with no cases of endophthalmitis out of 15,144 injections where similar strict aseptic measures were followed in an office setting [27].

Abell et al. [29] reported an endophthalmitis rate of 4/3,376 (0.12%) for office-based injections compared with 0/8,873 (0%) for OR-based injections. In this non-randomized series, patients with private health insurance were treated in the OR while those without insurance were treated in the office. The difference in endophthalmitis rates may be a reflection of socioeconomic or other factors [39]. Tabendeh et al. [30] reported an endophthalmitis rate of 3/8,210 (0.037%) anti-VEGF injections in the office compared with 2/3,047 in the operating room (0.066%), in another non-randomised study that was not powered to be able to detect a difference. Compared with office-based injections, there was no apparent benefit to an OR environment in this small study.

Although there is no doubt that the OR has many advantages, there are logistical hurdles that make access to OR facilities difficult for many patients, and the OR location can add substantial additional cost to patients and the healthcare system. It has been suggested that simply being in an OR may alter behavior with more careful attention to asepsis [23]. While three series describing OR-based injections have reported low rates of endophthalmitis, this may reflect publication bias. Similar low rates can be achieved with strict asepsis in an office setting.

### Hand antisepsis

The aim of surgical hand antisepsis is to reduce the bacterial load at the commencement of a procedure. Broadly speaking there are two main types of antisepsis solutions: aqueous scrubs (povidone iodine, chlorhexidine and triclosan) and liquid or gel alcohol rubs (with or without additional ingredients) [40].

Alcohol-based rubs have been found to have superior antimicrobial efficacy compared with aqueous scrubs [40, 41]. The reduction in microbial counts with alcohol rubs is rapid, and inhibition of bacterial regrowth to baseline levels can take more than 6 h [42]. However, unlike chlorhexidine, alcohol does not bind to the skin imparting a true residual effect, so chlorhexidine or other agents are often added to alcohol rubs [40].

While chlorhexidine induces less allergic reactions than povidone-iodine, skin irritation, dryness and irritant contact dermatitis still occurs more frequently with chlorhexidine scrubbing than with alcohol-based rubs [40, 43]. Liquid alcohol-based rubs are superior to gels both in terms of tolerability and efficacy [44].

Alcohol-based rubs are not without limitations. They do not remove surface dirt because they do not contain surfactants or have a foaming action, and may have limited effectiveness if the hands are heavily contaminated [40, 45]. Rubs may also leave a residue on the hands after use.

There is no specific evidence regarding the role of hand antisepsis in the context of intravitreal injection and most of the retrospective series do not even mention if or how hand antisepsis was performed. If sterile gloves are employed, the need for hand antisepsis could be questioned given the low risk of glove perforation during procedures of very short duration. In our opinion, attention to hand antisepsis is important for all invasive procedures in medicine. The initial antisepsis at the start of an injecting session should ideally include hand washing with soap or an aqueous scrub to mechanically remove any surface dirt or heavy bacterial contamination, especially if gloves are not worn [40, 46]. For subsequent antisepsis, alcohol-based rubs are ideal given their rapid action and superior dermal tolerance.

### Gloves

The purpose of wearing gloves during invasive procedures is to protect both the patient and the surgeon [47]. In a survey of retinal specialists in the USA, only 254/762 (33%) reported wearing sterile gloves for intravitreal injections, while 323/762 (42%) did not wear gloves at all [48]. In another smaller survey of 158 retinal specialists, 46% reported that they do not wear gloves [49]. In contrast, 88% of 112 surveyed retinal specialists in the United Kingdom reported using sterile gloves [50]. If non-sterile gloves are used, perforations may be more common with vinyl compared with latex gloves [51–53]. Non-sterile gloves may be more prone to fungal contamination compared with individually sealed sterile gloves [54].

It has been argued that sterile gloves are not required as long as the tip of any instrument touching the eye remains sterile [48, 55, 56]. By definition, this is no longer “aseptic technique”, a key principle of which is that any part of an instrument if touched directly or indirectly could result in infection [57]. Sterile gloves are required for aseptic procedures, while non-sterile gloves suffice for clean procedures [57]. A “no-touch” technique without gloves at all was advocated for cataract surgery over 50 years ago, but has fallen out of favor [58].

Wound infection rates have been shown to be no higher with the use of non-sterile compared with sterile gloves when suturing the skin [59, 60] However, sterile gloves are recommended for the insertion of central venous catheters and spinal anesthesia procedures [51, 61, 62]. Like the CSF, the vitreous is an immune-privileged site and a small inoculum of low virulence bacteria may be sufficient to cause endophthalmitis.

There are no studies directly examining the role of sterile gloves in reducing the risk of post-injection endophthalmitis, and they are often used in conjunction with other aseptic measures such as face masks and sterile drapes. We believe that intravitreal injection should be regarded as an aseptic procedure given that it involves penetration into an immune-privileged, nutrient-rich cavity. Similar to other aseptic procedures in medicine, the use of sterile gloves should be considered.

### Masks

The main purpose of wearing a surgical face mask is to reduce bacterial contamination of the surgical field from the surgeon’s mouth or nasopharynx [63]. In the 2013 ASRS PAT survey, just 14% of US-based ophthalmologists reported wearing a mask and asking the patient not to speak [34].

In his meta-analysis of 105,536 injections, McCannel found that eight of the 26 culture-positive cases (31%) were due to *Streptococcus* and noted that this was 3-fold higher than earlier studies of endophthalmitis after cataract surgery [8]. Others have found similar results and have highlighted the poor visual outcomes associated with this virulent pathogen, with an increased likelihood of a final VA of counting fingers or less and an increased likelihood of enucleation [14, 16, 17, 21, 64].

It is thought that the preponderance of Streptococcal isolates may relate to droplet dispersal of organisms while performing intravitreal injections. Viridans group *Streptococcus* species are normal commensals of the upper respiratory tract and oral cavity [65, 66]. As they are uncommonly found as part of the normal conjunctival flora, it has been suggested that they may contaminate the conjunctival surface or needle via aerosolization, leading to endophthalmitis [8, 21]. In studies where conjunctival cultures were taken in treatment-naïve eyes, the most commonly cultured organisms were coagulase negative Staphylococci accounting for 65–83% of isolates, while 0–7% of isolates were Streptococci [6, 67–69].

In an experimental study designed to simulate the conditions during intravitreal injections, Wen et al. [70] found that wearing a face mask or remaining silent significantly decreased culture plate contamination compared with not wearing a face mask or turning the face away. They also showed that a significant number of colonies grew when the reclined volunteer (simulating a patient) continued talking. While no Streptococcal species were isolated from the groups wearing a face mask or remaining silent, the proportion of bacterial colonies represented by oral Streptococcal species ranged from 67 to 83% in the other groups.

In a similar study, Doshi et al. [71] also found almost no bacterial growth when a mask was not worn and silence was not maintained, if the agar plates were pretreated with povidone-iodine (PI). In practice however, if the conjunctival surface were to be contaminated immediately prior to needle entry, there may not be adequate time for the PI to take effect.

Friedman et al. [72] recently demonstrated no difference in the needle contamination rates when speaking compared with maintaining silence.

Oral commensals have been found in cases of iatrogenic meningitis following dural puncture procedures and in some cases have been molecularly matched with strains found in the oropharynx of the proceduralist [73–76]. Absence of a face mask has also been implicated in iatrogenic septic arthritis after intraarticular injections [77, 78]. In these cases there is a strong suggestion that airborne transmission of a proceduralist’s oropharyngeal flora onto a needle or patient’s skin is followed by inoculation into a sterile compartment.

The presence of a viral upper respiratory tract infection (URTI) has been shown to markedly increase the airborne dispersal of methicillin-resistant *Staphylococcus aureus* (MRSA), which can be prevented by wearing a mask [79].

Several studies have shown that wearing a mask does not lower the risk of surgical wound infection [80–82]. Wen et al. argue that oral Streptococcal species are of relatively low virulence in the immunocompetent host, which is why they are infrequently found in surgical site infections. However the vitreous and cerebrospinal fluid are immune-privileged sites where these usually less virulent strains can flourish [70, 77, 83]. Dural puncture is a procedure similar to intravitreal injection in that both involve needle penetration into a nutrient-rich body cavity that can serve as a culture medium [8].

Of course, there may be other explanations for the preponderance of Streptococcal infections after intravitreal injections. Delayed-onset bleb-related endophthalmitis is also associated with a disproportionately higher rate of Streptococcal infection. This may be the result of alterations in the resident flora or structural changes in the eye wall, and such changes may also occur in some eyes after multiple intravitreal injections [30, 84–86].

While the use of face masks has not been proven to reduce the risk of post-injection endophthalmitis [87], they have been associated with a reduction in post-operative endophthalmitis [88]. Although maintaining total silence may be equivalent to the wearing of a mask, it is often important to give patients reassurance and instructions while performing the procedure [89–91]. A mask may also offer protection in the event of an inadvertent cough or sneeze. The needle should remain capped until immediately before the injection [21]. Patients should be instructed to minimize talking before or during the procedure. Assistants involved in setting up the instruments, drug and sterile trays should maintain silence or wear a face mask, and keep the trays covered until the commencement of the procedure. Patients’ relatives should be encouraged to wait outside the procedure room.

### Antisepsis

The aim of antisepsis is to reduce the bacterial load on the ocular surface and the periocular structures including the eyelids and eyelashes, without inducing antimicrobial resistance or selecting for more virulent organisms [92]. No study to date has found a correlation between the number of bacteria on the ocular surface and the risk of developing endophthalmitis [93]. Antibiotic resistance does not appear to impair the utility of PI and chlorhexidine, the two most commonly used antiseptics [94].

#### Povidone-iodine (PI)

PI is a complex of iodide and a solubilizing carrier, polyvinylpyrrolidone, which acts as a reservoir of “free” iodine, which is the active component [95]. The iodine penetrates bacterial cell membranes and inactivates key cytosolic proteins, fatty acids and nucleotides. PI does not have to be allowed to dry or evaporate to have a bactericidal effect [96]. It has a broad spectrum of antimicrobial activity with negligible bacterial resistance [97]. A recent survey found that over 99% of retinal specialists use PI before intraocular injections [48].

##### Efficacy

In a small randomized study, 5% PI instilled into the conjunctival sac prior to ophthalmic surgery reduced the number of bacterial colonies by 91%, compared with a 33% reduction in control eyes [98].

In a subsequent open-label non-randomized parallel trial, Speaker and Menikoff found that the incidence of culture-positive endophthalmitis was 0.06% in an operating suite using 5% PI, compared with 0.24% in another suite using silver protein solution (P < 0.03) [99].

##### Safety and toxicity

Adverse reactions to PI are usually the result of an irritant effect that is proportional to the duration of exposure, and consequently many patients report post-injection pain [100, 101]. A study in rabbits demonstrated significant epithelial fluorescein staining with 5% PI [102]. It should be irrigated thoroughly post-injection to minimize discomfort [100]. Less commonly, contact dermatitis may develop after repeated exposure [100, 101].

If PI is applied to the surface of the eye just before the needle is inserted through the pars plana, a small amount of PI may be introduced into the vitreous cavity [103]. Animal studies have shown that intravitreal injection of a small volume of low-concentration PI is well tolerated [104, 105].

Anaphylaxis to PI is rare, and there have been no reports of anaphylaxis following the topical ophthalmic use of PI [100, 103]. Furthermore, seafood allergy is not a contraindication to the use of topical PI, nor is reported allergy to iodinated contrast media [100]. Iodine is not the allergenic component of shellfish or contrast media, even though both contain iodine [100, 101, 106].

##### Method of instillation

A 10 mL flush of PI onto the conjunctival surface and fornices has been shown to lower conjunctival bacterial counts more than simply instilling a few drops [93, 107]. Flushing is thought to dislodge bacteria from the fornices, where the conjunctiva has many deep crypts, allowing PI to kill the organisms [93]. Selectively flushing one quadrant of the conjunctival surface while avoiding the cornea has not been compared with bathing the entire ocular surface. While PI may be applied to the eyelid margins and eyelashes, eyelid scrubbing should be avoided [108, 109].

##### Concentration and contact/kill-time

Half-strength (5%) PI is commonly used on the ocular surface to reduce its epithelial toxicity, but the most effective concentration is debatable [110].

Berkelman et al. [111] demonstrated that diluting full-strength (10%) PI paradoxically increased its bactericidal activity against *S. aureus* in vitro. After a 15 s exposure to PI, no organisms were recovered using concentrations of 0.1, 0.2 or 1% PI, but sterility was not achieved with such a short exposure using 5% or 10% PI. A 1–2 min exposure to 5% PI and a 4 min exposure to 10% PI was required to achieve sterility.

Ta et al. [112] found no difference in the conjunctival culture rates using 5% PI for 1 min, compared with 10% PI for 5 min. Van Rooij et al. [113] reported no increase in their rate of post-cataract surgery endophthalmitis, after switching from 5 to 1% PI. Shimada et al. [27] described a series of 15,144 injections using 0.25% PI, without a single case of endophthalmitis.

In contrast, using a 2-min contact time, Ferguson et al. [110] found that 5% PI was more effective than 1% PI at reducing the number of colony forming units, particularly in the presence of a heavier initial bacterial load. While the concentration of free iodine may be higher at lower concentrations of PI, it is thought that a lower concentration of PI has a lower reservoir of available iodine, which is exhausted when the bacterial load is increased.

Friedman et al. [114] found a significant reduction in conjunctival bacterial growth after a 30 s exposure to 5% PI, and a further reduction with a 60+ second exposure, while 15 s was inadequate.

##### Post-injection antisepsis

Post-injection PI may reduce the risk of subsequent bacterial entry via a “vitreous wick” through the wound track. The role of this potential mechanism for endophthalmitis is unclear. Apt et al. [115] showed that 5% PI instilled at the conclusion of cataract surgery suppressed bacterial growth more than an antibiotic solution, for 24 h following surgery. Shimada et al. [27] used a 5 mL flush of 0.25% PI before and after injections with an endophthalmitis rate of 0/15,144.

##### Resistance

Hsu et al. recently showed that bacterial resistance does not develop in patients undergoing serial intravitreal injections with povidone-iodine preparation alone, without the use of pre- or post-injection topical antibiotics, confirming previous in vitro work [116–118].

#### Aqueous chlorhexidine

Chlorhexidine is a cationic biguanide that damages the outer bacterial surface layers and subsequently attacks the cytoplasmic membrane of the organism [95]. Chlorhexidine, like PI, is a broad-spectrum antimicrobial agent. Compared with PI, chlorhexidine may act less rapidly, but exhibits sustained antimicrobial activity, and is not readily neutralized by organic matter [95, 119].

##### Efficacy

The mean reduction in conjunctival bacterial counts prior to corneal suture removal using a 3 min exposure to 4% PI was 91%, compared with 88% using 0.05% chlorhexidine, a non-statistically significant difference [120]. In another study, there was no difference in the culture rate from conjunctival swabs in patients receiving either 0.05% chlorhexidine or 0.6% PI [121].

In a multi-center retrospective study where aqueous chlorhexidine gluconate 0.1% was used as the sole antiseptic measure prior to intravitreal injection, the endophthalmitis rate was 3/40,535 (0.0074%; 1 in 13,512) [Personal communication, Dr Peter Davies, Newcastle, NSW Australia].

##### Safety and toxicity

The safety of aqueous chlorhexidine has been detailed in numerous animal studies [122–124], however alcohol-based or detergent-based chlorhexidine causes severe corneal epithelial toxicity [125]. Topical chlorhexidine 0.02% to 0.2% is used as a treatment for acanthamoeba keratitis [126]. While endothelial toxicity is well described, a significant concentration of chlorhexidine is unlikely to reach the endothelium after intravitreal anti-VEGF injection [127–129].

##### Resistance

The acquisition of resistance to chlorhexidine has been demonstrated in vitro but whether it occurs on the ocular surface after repeated intravitreal injections is yet to be studied [117]. The reduced susceptibility of methicillin-resistant *S. aureus* (MRSA) to chlorhexidine compared with methicillin-sensitive *S. aureus*, and the presence of efflux-mediated resistance genes in staphylococci is of concern [130].

#### Povidone-Iodine + chlorhexidine

Synergy when using both PI and chlorhexidine has been described in vitro and in clinical studies on the skin, but has not been studied on the eye [95, 131, 132].

#### Saline irrigation alone

Irrigation with saline alone has been shown to be ineffective in reducing the conjunctival bacterial load and may even increase bacterial counts, perhaps because bacteria are dislodged from the fornices but not subsequently killed. [114, 133, 134]

In summary, the optimal concentration and contact-time for antiseptic agents likely depends on the bacterial load, the spectrum of bacteria present and their virulence, and the robustness of the host defense mechanisms. While a longer contact time may lead to a greater reduction in the bacterial load, it may be complicated by more epithelial toxicity. With either agent, our practice is to copiously irrigate the ocular surface with normal saline after the procedure, to minimize epithelial toxicity. For patients who have experienced severe pain after an injection we have recommended they use preservative-free lubricants for a day prior to their subsequent injections with good effect.

### Anesthesia

Anesthesia prior to intravitreal injection can be achieved using topical drops (alone or on a sterile cotton-tipped applicator or pledget), viscous gel, subconjunctival injection or a combination of these [135, 136]. Drops are typically used prior to gel or subconjunctival injection.

#### Viscous gel anesthesia

Lidocaine gel has been shown to act as a barrier preventing PI from coming into contact with bacteria on an agar plate [137], and was shown to be a risk factor for post-operative endophthalmitis if applied prior to PI [138]. No cases of endophthalmitis were found in a series of 4,690 injections where PI was instilled both before and after application of lidocaine 2% gel [139]. In vitro, application of PI for a mere 5 s prior to lidocaine gel application has been shown to be effective in inhibiting bacterial growth [140].

In contrast, Dahl et al. [141] found reduced colony forming units in eyes when lidocaine 2% gel was applied before PI, compared with when PI was used alone (P = 0.08). Lidocaine gel plated with *S. aureus* and *Escherichia coli* showed a ring of inhibition to both bacteria, suggesting that lidocaine gel might independently exert an antimicrobial effect. Lad et al. found no difference in the post-injection endophthalmitis rate when lidocaine gel was applied prior to PI (4/4,682; 0.085%), compared to when PI was used alone (4/4,120;0.097%) (P = 1.00) [142]. Although gel may act as a complete barrier to antiseptic agents on an agar plate, the ocular surface is warm, has continuous tear production and there is movement between the globe and the eyelid, all of which aid the dispersal of gel.

#### Subconjunctival anesthesia

In a retrospective series, Tustin et al. reported a rate of endophthalmitis of 8/8,189 (0.1%) when subconjunctival lidocaine was not used, compared with 0/6,853 when it was used (P = 0.03), suggesting that the use of subconjunctival anesthetic may reduce the risk of endophthalmitis [143]. However, 14 culture-negative cases of “possible” endophthalmitis were excluded, nine of which were after injection of triamcinolone and five after bevacizumab. The same authors found that 2% lidocaine/0.1% methylparaben demonstrated rapid bactericidal effects against *S. aureus*, *S. epidermidis* and *S. viridans* in vitro [143].

If antiseptics are not used prior to administration of subconjunctival anesthetic, surface bacteria may be introduced into the subconjunctival space by the needle, and these could in turn be introduced into the vitreous cavity [144]. Nonetheless, Tustin et al. [143] only applied PI after the subconjunctival injection of anesthetic, and not before, with no cases of definite endophthalmitis.

While lidocaine may have inherent bactericidal properties, the reduction in endophthalmitis risk with subconjunctival lidocaine that Tustin et al. reported may be the result of other factors. The bleb of anesthetic may help to disconnect the vitreous body from the conjunctival surface, or dilute the pathogens that are present. As subconjunctival anesthesia works best if left for at least a few minutes, it allows the povidone-iodine to remain in contact with the ocular surface for longer than usual. Finally, adequate anesthesia is crucial so that the patient does not move or squeeze their eyes as the needle enters the eye, as these sudden involuntary responses can compromise the sterility of the needle. With the deeper anesthesia afforded by subconjunctival injection, this may be another explanation for their results.

The application of antiseptic agents before and after gel or subconjunctival anesthetic is important, and any intrinsic antimicrobial activity of lidocaine should not be relied upon. When gel anesthesia is used, the authors remove any residual gel by rolling a sterile cotton-tipped applicator over the conjunctival surface prior to flushing with more PI.

### Antibiotics

In the ASRS PAT Surveys the percentage of respondents using pre-injection antibiotics was 40% in 2008, 39% in 2009 and 27% in 2011 [34]. The percentage using them post-injection was 86% in 2008, 82% in 2009 and 62% in 2011. In 2013, 78% of US respondents indicated no use of pre- or post-injection antibiotics.

#### Pre-injection antibiotics

There have been no prospective studies showing that pre-injection antibiotics reduce the risk of endophthalmitis. Isenberg et al. reported synergy between antibiotics and PI, with sterile conjunctival cultures found in 31% of eyes treated with a 3 day course of pre-operative antibiotics alone and in 40% treated with PI alone, compared with sterile cultures in 83% of eyes that received both antibiotics and PI [145]. However, given the longer kill-time of antibiotics compared with PI, using antibiotics just 1–2 h pre-operatively conferred no additional benefit over PI alone in two studies [146, 147].

In a study of patients undergoing regular intravitreal injections, the rate of positive bacterial cultures was 8% in the group that received a 3 day course of pre-injection gatifloxacin in addition to PI, compared with just 4% in the group that received PI alone (P = 0.32) [69]. The lack of synergy may be a reflection of increased antibiotic resistance in patients having regular injections with repeated antibiotic exposure, as compared with patients about to undergo cataract surgery who are relatively antibiotic naive.

#### Post-injection antibiotics

Antibiotics have been used post-injection, but not pre-injection, in several series without the prevention of endophthalmitis (see Table 1) [17, 19, 22, 25, 26, 31, 32]. In fact, a non-statistically significant higher rate of endophthalmitis has been found in patients receiving post-injection antibiotics in a number of studies [16, 28, 148–150].

#### Antibiotic resistance

Coagulase-negative Staphylococcus endophthalmitis isolates resistant to fluoroquinolones at Bascom Palmer Eye Institute increased from 0 to 10% in 1990–1994, to 17–21.8% in 1995–1999, to 26.9–38.4% in 2000–2004, to 57.8–60.5% in 2005–2011 [151–153]. It is suspected that the widespread use of fluoroquinolones is responsible for the increasing resistance.

In 2010, the Antibiotic Resistance of Conjunctiva and Nasopharynx Evaluation (ARCaNE) investigators reported a significant baseline level of bacterial resistance in the conjunctiva of patients with newly diagnosed choroidal neovascularisation [67]. They subsequently showed that repeated intermittent exposure of ocular flora to topical antibiotics selected for resistant strains, which emerged immediately after exposure to antibiotic, and were maintained by periodic re-exposure [154, 155]. Co-resistance to other antibiotics also developed [156].

In another study, eyes receiving a 4-day course of fluoroquinolones after each injection were found to have a resistance rate of 87.5% compared with 25% in control eyes [157]. In a larger prospective study, a 3-day course of topical moxifloxacin after each injection increased the percentage of resistant isolates from 0% at baseline to 50% at month three [68].

In an animal study of experimentally-induced *S. epidermidis* endophthalmitis, antibiotic-resistant strains caused more inflammation and destruction of the infected retina compared with antibiotic-susceptible strains [158]. The same authors also noted that only antibiotic resistant strains of *S. epidermidis* have been isolated from their patients with post-operative endophthalmitis. It is postulated that antibiotic resistance leads to colonization by more virulent bacterial strains that can overcome the host defense mechanisms more easily, resulting in a higher likelihood of endophthalmitis [28, 159]. Antibiotic-resistant strains may also be more difficult to treat.

#### Antibiotic penetration

It has been shown that the topical administration of second-generation (ciprofloxacin, ofloxacin) and fourth-generation (moxifloxacin, gatifloxacin) fluoroquinolones leads to effective levels in the aqueous but not in the vitreous, in the non-inflamed eye [160, 161].

In summary, topical antibiotics not only have minimal vitreous penetration and seem to be unhelpful in preventing endophthalmitis, but with increasing resistance and selection of more virulent strains, routine antibiotic use could be harmful and we do not recommend their use either pre- or post-injection. Pre-injection topical antiseptics must be used, and to alleviate post-injection discomfort, patients can use preservative-free lubricants.

### Speculum

It is critically important to avoid contaminating the needle with the eyelashes or lid margins before or during entry into the globe, as direct inoculation is considered to be the major mechanism by which endophthalmitis occurs [108, 162]. A speculum is a reliable way to isolate the injection site, but many patients find them uncomfortable.

In a survey of retinal specialists in the USA, 92% of respondents stated that they routinely use an eyelid speculum to keep the eyelashes away during intravitreal injections [48]. A closed-blade speculum is superior to an open-blade speculum as it covers the eyelashes more effectively [163], but the most temporal lashes may remain exposed with any speculum. In some patients, excessively long eyebrow hairs also need to be avoided. In eight of the 12 patients with endophthalmitis in the VISION study, the infection was associated with protocol violations, the most common being failure to use an eyelid speculum [162].

Insertion of a speculum could theoretically squeeze secretions and bacteria out of the Meibomian glands, particularly in patients who forcefully squeeze their eyelids against the speculum. For this reason, it has been recommended that further PI should be instilled after speculum insertion [35, 108]. In a randomized controlled study, Friedman et al. [114] recently showed that the placement of a lid speculum did not in fact increase the number of conjunctival bacterial colony forming units.

Tailor et al. [164] found that the insertion of a lid speculum was the third most uncomfortable step during an intravitreal injection. Fineman et al. [32] found that a two-person bimanual eyelid retraction technique has the advantage of less patient discomfort. In their series of 10,164 injections without the use of a speculum there were three cases of endophthalmitis (0.03%). One-person bimanual retraction techniques using the fingers [165], a cotton-tip applicator [166], or a Desmarres lid retractor [167] have recently been described. In the PIE study, the endophthalmitis rate was 13/12,500 (0.10%) with the use of a bladed lid speculum, compared with 10/15,236 (0.066%) without (P = 0.27) [168].

While the experience in the VISION study made a compelling argument for the use of eyelid speculums, more recent evidence suggests that alternative methods of isolating the eyelids and eyelashes may be acceptable. With or without a speculum, we believe that if a needle has inadvertently touched anything but the bulbar conjunctiva at the injection site before or during entry into the eye it should be immediately withdrawn and discarded to avoid inoculating the vitreous. While PI may be applied to the eyelid margins and eyelashes, the adnexae should still be considered non-sterile.

### Sterile drapes

In a survey of retinal specialists in the USA, 668 of 759 respondents (88%) reported not using a sterile drape [48]. It has been suggested that a sterile adhesive drape isolates the patient’s nose and oropharynx from their eye, and theoretically could reduce bacterial aerosolization originating from the patient [19, 70].

In a study evaluating patients’ experiences at different stages of the injection procedure, the application, cutting and removal of an adhesive drape were found to be the most uncomfortable aspects of the procedure, bar the injection itself [164].

Sterile drapes allow the physician to position the patient’s head without contaminating their sterile gloves. It is unclear how important they are in reducing the risk of endophthalmitis given that their use is often associated with other aseptic measures including the use of sterile gloves and face masks.

### Techniques to minimize vitreous reflux

When an intravitreal injection needle is withdrawn from the eye, a subconjunctival bleb forms in approximately one-third of cases [169]. It is thought to comprise liquefied vitreous as well as some of the injected drug [170–172], but the presence of reflux does not lead to a subtherapeutic effect [173]. There is a theoretical risk of endophthalmitis occurring when organisms gain entry into the vitreous cavity through a “vitreous wick” [174]. Turgut et al. [175] found less vitreous reflux with injections performed through the inferotemporal quadrant compared with the superotemporal quadrant.

Displacement of the conjunctiva with a sterile cotton tip applicator before injection is thought to provide a disconnect between the vitreous cavity and the external eye, keeping the vitreous wick subconjunctival [35]. In the PIE study this technique was not associated with a reduced risk of endophthalmitis [168]. Inoculation of the vitreous with a cotton fiber has been reported as a complication [176, 177]. Applying pressure to the injection site immediately after needle withdrawal has also been advocated to help reduce the amount of vitreous reflux [174].

Tunneled injections through the sclera are associated with less vitreous reflux compared with straight injections that enter the sclera perpendicularly [178–180]. The most commonly used needle size for anti-VEGF injections is 30G [48]. 31G and 32G needles have been shown to produce less vitreous reflux in patients [181, 182] and in live rabbits [170], however in one cadaveric study [183] more reflux was found with 32G compared with 30G needles.

The reduced vitreous reflux with 32G needles or tunneled injections is associated with a higher immediate, though transient, intraocular pressure (IOP) elevation [179, 180, 184, 185]. Hoang et al. [184] have reported that repeated intravitreal injections in an eye may be complicated by sustained elevations in IOP. They have discussed possible mechanisms by which long-term ocular hypertension could result, and have hypothesized that repeated injections with minimal reflux may cause mechanical expansile stress on the trabecular meshwork.

In summary, techniques to minimize vitreous reflux have not been proven to reduce the risk of endophthalmitis and may be associated with more ocular hypertension.

### Disinfection of drug vials

The pharmaceutical companies and the Centre for Disease Control recommend that the rubber diaphragm of a drug vial should be disinfected with alcohol prior to drawing medication [186].

Buckley et al. [187] found that the diaphragm of one single use vial was contaminated with *Staphylococcus epidermidis,* while the other 99 they examined were sterile. Hilliard et al. [188] reported contamination of the diaphragm in two out 12 vials, despite an intact dust plastic cover.

In short, disinfection of the rubber diaphragm of vials with an alcohol swab is recommended, but the use of pre-filled syringes eliminates this step.

### Same day bilateral Intravitreal Injections

For patients with bilateral disease requiring relatively frequent dosing, bilateral same day injections can reduce the burden of treatment. In the 2013 ASRS PAT survey 50% of respondents reported doing same day bilateral injections compared with only 27% in 2008 [34].

Tabatabaii et al. [189] have reported two cases of bilateral endophthalmitis following bevacizumab, resulting in 20/400 vision bilaterally in one patient and light perception bilaterally in another.

Pooling the results of eight series, out of 3708 episodes of bilateral anti-VEGF injections (7,416 injections), there have been three cases of unilateral culture-positive endophthalmitis, one case of unilateral culture-negative endophthalmitis, and one case of unilateral intraocular inflammation [190–197].

Numerous outbreaks of endophthalmitis after injection with compounded bevacizumab have been reported, and are thought to be due to contamination during syringe preparation at the compounding pharmacy. Goldberg et al. [198] have suggested using bevacizumab syringes from two different batches for bilateral same-day injections.

Given the low risk, bilateral injections appear appropriate in select patients. While bilateral injections should be considered as two separate procedures, with separate instruments and ideally drug from different lots, they cannot be truly independent given that the patient is likely to have similar risk factors in both eyes, for example similar conjunctiva flora [198–200]. The surgeon is likely to employ similar aseptic measures and technique in both eyes and in many centers only one procedure room is available.

## Conclusion

Each step of the intravitreal injection procedure has been examined, and the relative importance of each aspect in lowering the risk of endophthalmitis can be debated at length.

From the available evidence, we believe that antibiotics pre- or post-injection should be omitted, placing further importance upon the need for adequate pre-injection antisepsis, which should be applied both before and after gel or subconjunctival anesthetic. We advocate the wearing of face masks or maintaining silence given the risk of aerosolization of bacteria. Exposure of the injection site can be achieved with or without a speculum and the needle must not make contact with anything before entering the eye. Adhering to strict aseptic technique in an office or operating room may help achieve the goal of lowering the risk of endophthalmitis following intravitreal injection.

The major weakness of this review is that it relies heavily upon retrospective data with the inherent selection biases of such studies. Prospective studies will allow firmer recommendations to be made in the future.

## Abbreviations

VEGF: vascular endothelial growth factor
OR: operating room
ASRS: American Society of Retinal Specialists
PAT Survey: Preferences and Trends Survey
PI: povidone-iodine
IOP: intraocular pressure
